# Trends in Diabetic Retinopathy, Visual Acuity, and Treatment Outcomes for Patients Living With Diabetes in a Fundus Photograph–Based Diabetic Retinopathy Screening Program in Bangladesh

**DOI:** 10.1001/jamanetworkopen.2019.16285

**Published:** 2019-11-27

**Authors:** Mahiul M. K. Muqit, Nick Kourgialis, Meredith Jackson-deGraffenried, Zaman Talukder, Erica R. Khetran, Arifur Rahman, Weng Onn Chan, Fakrhul A. Chowdury, Dipak Nag, Jasmin Ahmad, David S. Friedman

**Affiliations:** 1 Vitreoretinal Service, Moorfields Eye Hospital, National Health Service Trust, London, United Kingdom; 2 Institute of Ophthalmology, University College London, London, United Kingdom; 3 Helen Keller International, New York, New York; 4 Helen Keller International, Dhaka, Bangladesh; 5 Department of Ophthalmology and Visual Sciences, University of Adelaide, Adelaide, South Australia, Australia; 6 Retina Service, National Institute of Ophthalmology and Hospital, Dhaka, Bangladesh; 7 Retina Service, Chittagong Eye Infirmary and Training Complex, Chittagong, Bangladesh; 8 Massachusetts Eye and Ear, Harvard Medical School, Boston

## Abstract

**Importance:**

Diabetic retinopathy (DR) is the leading cause of low vision among working-age adults. An estimated 6.9 million people in Bangladesh were living with diabetes in 2017, which is projected to increase to more than 10 million people in 2025. Currently, no standardized and/or large-scale DR screening program exists in Bangladesh.

**Objective:**

To develop a novel fundus photograph–based eye screening model for early detection of DR to prevent vision loss in Bangladeshi individuals with diabetes.

**Design, Setting, and Participants:**

In this cross-sectional study, 49 264 patients with diabetes underwent opportunistic eye screening at 2 eye hospitals and 1 diabetic hospital in Bangladesh between June 1, 2010, and September 30, 2017. The data set was analyzed from April 8 to December 30, 2018. Technicians were trained to obtain 2-field digital fundus photographs and to grade each according to a standardized DR severity scale. Each patient was counseled and triaged for treatment using defined DR referral criteria.

**Main Outcomes and Measures:**

Primary DR grading outcomes, visual acuity, and treatment outcomes.

**Results:**

A total of 49 264 patients (54.3% male; mean [SD] age, 50.8 [12.3] years) underwent DR screening during a 7-year period. The DR prevalence rate across all 3 sites was 33% (95% CI, 33%-33%). Prevalence rates varied by center (Chittagong, 64.6% [95% CI, 64.0%-65.0%]; Dhaka, 39.8% [95% CI, 39.0%-41.0%]; and Feni, 13.0% [95% CI, 13.0%-14.0%]). Across all age groups, male patients were at higher risk of prevalent DR than female patients (odds ratio, 1.99; 95% CI, 1.90-2.07). The prevalence was 3.9% for preproliferative DR, 7.8% for proliferative DR, and 19.2% for maculopathy. Individuals with DR had significantly worse visual acuity than those with no DR (best-corrected visual acuity, 0.35 vs 0.21 logMAR; *P* < .001). The rate of moderate visual impairment was 12.2%, and the rate of blindness was 2.5%. Primary treatments included laser photocoagulation (n = 1637), intravitreal injection (n = 1440), and vitrectomy (n = 309).

**Conclusions and Relevance:**

Screening Bangladeshi individuals known to have diabetes using fundus photography identified large numbers of patients with sight-threatening proliferative DR, maculopathy, and visual impairment or blindness. Expansion of eye screening services in Bangladesh is warranted as part of a national government eye care and diabetes health policy.

## Key Points

**Question:**

Is it feasible to develop a systematic model of fundus photograph–based eye screening for patients living with diabetes in Bangladesh?

**Findings:**

In this cross-sectional study, 49 264 patients underwent fundus photograph–based eye screening during a 7-year period at 3 centers in Bangladesh. The primary prevalence rate of diabetic retinopathy was 33%.

**Meaning:**

Given the very high prevalence of diabetes, there is an urgent need to scale up diabetic eye screening services in resource-poor countries to prevent retinopathy complications and vision loss.

## Teaser

This cross-sectional study examines a novel fundus photograph–based eye screening model for early detection of diabetic retinopathy to prevent vision loss in Bangladeshi individuals with diabetes.

## Introduction

Type 1 and type 2 diabetes is rapidly increasing as a major cause of morbidity and mortality globally.^1,2^ According to the World Health Organization and the International Diabetes Federation, the number of people living with diabetes was 425 million in 2017 and is projected to increase to 629 million in 2045.^3^ The International Diabetes Federation estimated that 6.9 million people in Bangladesh were living with diabetes in 2017, increasing to more than 10 million in 2025.^3^

VISION 2020: The Right to Sight initiative lists diabetic retinopathy (DR) as one of its priority eye diseases for Southeast Asia and other regions.^4^ Diabetic retinopathy is a leading cause of early-onset blindness among working-age people with diabetes worldwide.^5^ It affects nearly all individuals with type 1 diabetes and approximately 77% of those with type 2 diabetes who have had diabetes for 20 years or longer.^6^ The risk of DR is directly associated with the duration of individual diabetes cases and the level of glucose control maintained.^5^

Bangladesh has among the world’s largest number of people with diabetes, with 35% of the population older than 35 years receiving a diagnosis of diabetes or prediabetes.^7,8^ The public and private health care systems in Bangladesh lack effective models for implementing DR screening programs at the national level. The enormous screening burden cannot be effectively addressed by using DR screening clinics within eye hospitals alone. The current system fails to raise awareness and motivate patients to seek regular screening for DR, and almost no organizations are systematically engaged in screening for DR.

Diabetic retinopathy is asymptomatic until the later, more severe stages, but retinal screening can dramatically reduce the risk of blindness because treatment of retinopathy at an early stage allows for good vision to be retained.^9^ With proper screening and treatment, almost all blindness from DR can be prevented. We present the findings from a DR screening program focused on identifying DR among individuals presenting for diabetes care in Bangladesh.

## Methods

### Population

The patients in this cross-sectional study were individuals with diabetes who were screened at 3 different hospitals in Bangladesh between June 1, 2010, and September 30, 2017. All data were collected at the 2 participating eye hospitals—Chittagong Eye Infirmary and Training Complex (CEITC) and the National Institute of Ophthalmology (NIO) and Hospital—as well as at Feni Diabetes Hospital (FDH). Ethical approval for the projects was granted by the local institutional review boards at each institution, with a memorandum of understanding established between Helen Keller International and each institution, and study ethical approval granted by John Hopkins Hospital Institutional Review Board. All eligible patients provided written informed consent to undergo DR screening as part of standard hospital policy. This study was performed according to Strengthening the Reporting of Observational Studies in Epidemiology (STROBE) guidelines.^10^

This project had 2 stages. In the first stage, a DR screening service was set up in 2 locations in 2009, a center based in an eye hospital (that included the reading center where the retinal images from the FDH and CEITC were graded by the team) at the CEITC and a screening center at the FDH. In both centers, people with diabetes were invited to undergo fundus photography–based DR screening. A 3-tier workforce was established that included primary and secondary nonmedical photographic graders with ophthalmologists (D.N. and J.A.) as arbitration graders. The education and counseling of the patients and the training of the screening team were coordinated by Helen Keller International with local health care professionals. Treatment pathways included laser, intravitreal injection therapy, and vitreoretinal surgical services. A licensed electronic medical record known as OptoMIZE (Emis Health) approved for DR screening was used to capture patient data and fundus photographs and to manage the patient workflow and triage for graders and ophthalmologists.

The second stage of the DR screening project involved a scaling up of the DR screening services in Dhaka, the capital city of Bangladesh. This second stage began in 2014 and was based in the Government of Bangladesh NIO and Hospital, which is the largest government tertiary eye care center in Bangladesh.

The screening locations all had a patient waiting area, a photography area equipped with a fundus camera, a grading area, and a dedicated space for counseling and health education. These clinics were set up within the retinal outpatient zone of each hospital.

### Procedures

The DR screening clinics offered a “walk-in” system whereby patients with diabetes were referred from the eye clinics on the same day within the FDH, NIO, and CEITC, and patients were directed to the DR screening clinics. Two standard fundus photographs with dilated pupils—fovea centered and disc centered—were taken of each eye using a table-mounted fundus camera (Topcon TRC-NW8; Topcon Corp). These photographs were graded by primary and secondary graders according to a standard retinopathy (R) and maculopathy (M) grading classification.^11^ The images were transmitted electronically to a grading area according to the primary screening location. The outcomes of DR grading include no retinopathy (R0), mild retinopathy (R1), preproliferative retinopathy (R2), proliferative DR (PDR), no maculopathy (M0), and referable maculopathy (M1). Medical retinal specialist ophthalmologists arbitrated fundus photographs when the graders disagreed. The graders assessed the images while the patient waited, and grading outcomes were then triaged on the OptoMIZE electronic medical record. Through a team of designated health educators and counselors, patients were provided with the DR screening results and advised on recommended follow-up.

### Training of Graders

We developed a 3-tier grading team that involved a nonmedical workforce of nurses, optometrists, and paramedics for primary and secondary fundus photograph grading. Only medical retina specialist ophthalmologists performed arbitration grading. This grading model was based on the National Health Service Diabetic Eye Screening Programme (NHS DESP) used in England.^11^ Graders were trained through workshops and on-site practical sessions using standardized materials from the NHS DESP and Moorfields Eye Hospital. The staff responsible for undertaking DR screening and grading included ophthalmic nurses, paramedics, and nurses and ophthalmologists. All staff underwent appropriate training and local accreditation by a retina specialist from Moorfields Eye Hospital that included the following core and mandatory training modules: (1) national screening program: principles, processes, and protocols; (2) diabetes and its relevance to retinopathy screening including the anatomy, physiology, and pathologic characteristics of the eye and its clinical relevance; and (3) detecting retinal disease and classifying DR.

### Quality Assurance Standards and Fail-safe

During photographic grading by primary graders, there was an automated internal quality assurance system whereby 10% of normal R0M0 photographs graded by the primary grader were automatically sent for secondary grading. A monthly online tool was developed for all DR screening graders to be assessed using an international version of the test and training module, as is conducted in the UK NHS DESP. A retina specialist from Moorfields Eye Hospital conducted external quality assurance annually during each project. Internal quality assurance was conducted by the medical retina specialist at each center, and individualized training was provided to improve education and training for DR grading. As part of the program, the medical retina specialists received training on the latest laser photocoagulation guidelines by a retina specialist from Moorfields Eye Hospital. During the program, a fail-safe officer from Helen Keller International monitored the technical, screening, and counseling activities to ensure optimal delivery. To reduce patient risk, all patients with positive screening results who were logged as requiring eye treatment were monitored as part of internal quality assurance. This method also served to identify any barriers or delays in treatment.

### Outcomes

The primary objective of this study is to describe the grades of DR detected from fundus photograph–based eye screening for patients with diabetes in Bangladesh. The secondary objectives were to explore the presenting visual acuity (VA) levels in the population with DR across the 3 regions in Bangladesh and their association with vision loss. Patient data were evaluated at quarterly intervals for the duration of the project to ensure that only new cases of DR were captured for this study. Treatment outcomes for laser photocoagulation, intravitreal anti–vascular endothelial growth factor injections (intravitreal injections), and vitrectomy were validated against hospital logbooks to ensure that the first treatment outcome was captured. The World Health Organization classification for visual impairment comprises categories 0 to 5; blindness comprises categories 3 to 5. Mild or no visual impairment (level 0) is indicated by a VA 6/6 to 6/18, moderate visual impairment (level 1) is indicated by a VA greater than 6/18 to 6/60, severe visual impairment (level 2) is indicated by a VA greater than 6/60 to 3/60, and blindness (levels 3, 4, and 5) is indicated by a VA of greater than 3/60 to no light perception.^12^

### Statistical Analysis

The data set was analyzed from April 8 to December 30, 2018. This article presents the data from a screening program, and no statistical inference to the general population was made. Summary statistics were calculated for descriptive variables, and analysis of variance with Tukey post hoc multiple comparison adjustment was used to compare mean values of best-corrected VA within groups. Exploratory analysis was performed to identify correlates in the screened population. The odds ratio was calculated from a contingency table and binomial logistic regression. Multivariate logistical analysis was used to evaluate the associations of age and sex with DR. A descriptive analysis of treatment outcomes was performed. For analyses, *P* values were from 2-sided tests, and results were deemed statistically significant at *P* < .05. All analyses were performed using SPSS Statistics for Windows, version 25.0 (IBM Corp).

## Results

A total of 49 264 patients (45.7% female and 54.3% male; mean [SD] age, 50.8 [12.3] years) underwent DR screening during a 7-year period. Combining data across the 3 locations yielded a total of 49 209 patient records (55 cases with missing data were excluded) (Table). Between June 1, 2010, and September 30, 2017, 13 189 individuals attended the CEITC and 23 959 individuals attended the FDH for DR screening. Between November 1, 2014, and August 31, 2017, 12 061 individuals attended the NIO for DR screening. The mean (SD) age was 54.1 (10.1) years for those screened at the CEITC (range, 12-99 years), 48.1 (13.6) years for those screened at the FDH (range, 10-110 years), and 52.4 (10.6) years for those screened at the NIO (range, 11-116 years). A total of 30 107 of 45 021 images (66.9%) were graded R0M0 with no DR present. There were 4243 ungradable images owing to cataract in most eyes. However, the cause for an ungradable image was not adequately logged in the system; therefore, the reasons for the poor-quality images are uncertain.

**Table.** Demographic Characteristics of Age, Sex, and Diabetic Retinopathy Grade

Characteristic	CEITC	FDH	NIO	Total, No./Total No. (%)
Age, mean (SD), y	54.1 (10.1)	48.1 (13.6)	52.4 (10.6)	50.8 (12.3)
Sex, No./total No. (%)				
Male	6760/11 567 (58.4)	9972/22 141 (45.0)	8132/12 061 (67.4)	24 864/45 769 (54.3)
Female	4807/11 567 (41.6)	12 169/22 141 (55.0)	3929/12 061 (32.6)	20 905/45 769 (45.7)
Presence of diabetic retinopathy, No./total No. (%)				
Normal	4103/13 189 (31.1)	19 359/23 959 (80.8)	6645/12 061 (55.1)	30 107/49 209 (61.2)
Diabetic retinopathy	7486/13 189 (56.8)	2978/23 959 (12.4)	4395/12 061 (36.4)	14 859/49 209 (30.2)
Ungradable	1600/13 189 (12.1)	1622/23 959 (6.8)	1021/12 061 (8.5)	4243/49 209 (8.6)
Worst grade of retinopathy, No./total No. (%)				
R0	4103/13 189 (31.1)	19 359/23 959 (80.8)	6645/12 061 (55.1)	30 107/49 209 (61.2)
R1	4165/13 189 (31.6)	2353/23 959 (9.8)	2612/12 061 (21.7)	9130/49 209 (18.6)
R2	1210/13 189 (9.2)	311/23 959 (1.3)	392/12 061 (3.3)	1913/49 209 (3.9)
R3	2111/13 189 (16.0)	314/23 959 (1.3)	1391/12 061 (11.5)	3816/49 209 (7.8)
Ungradable	1600/13 189 (12.1)	1622/23 959 (6.8)	1021/12 061 (8.5)	4243/49 209 (8.6)
Worst grade of maculopathy, No./total No. (%)				
M0	7093/13 189 (53.8)	21 044/23 959 (87.8)	7388/12 061 (61.3)	35 525/49 209 (72.2)
M1	4496/13 189 (34.1)	1293/23 959 (5.4)	3652/12 061 (30.3)	9441/49 209 (19.2)
Ungradable	1600/13 189 (12.1)	1622/23 959 (6.8)	1021/12 061 (8.5)	4243/49 209 (8.6)

Abbreviations: CEITC, Chittagong Eye Infirmary and Training Complex; FDH, Feni Diabetes Hospital; M, maculopathy; NIO, National Institute of Ophthalmology; R, retinopathy.

**Figure 1.**
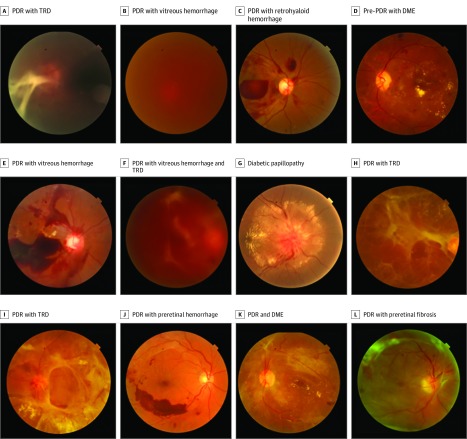
Examples of Disc-Centered and Macula-Centered Screening Photographs A, Proliferative diabetic retinopathy (PDR) with tractional retinal detachment (TRD) (retinopathy [R]3). B, PDR with vitreous hemorrhage (R3). C, PDR with retrohyaloid hemorrhage (R3). D, Pre-PDR with diabetic macular edema (DME) (R2 maculopathy [M]1). E, PDR with vitreous hemorrhage (R3). F, PDR with vitreous hemorrhage and TRD (R3). G, Diabetic papillopathy. H, PDR with TRD (R3). I, PDR with TRD (R3). J, PDR with preretinal hemorrhage (R3). K, PDR and DME (R3M1). L, PDR with preretinal fibrosis (R3).

**Figure 2.**
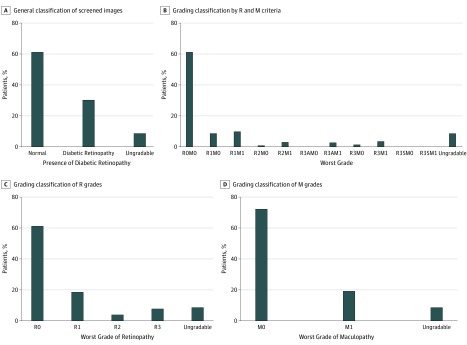
Total Proportion of Diabetic Retinopathy Screening According to Retinopathy and Maculopathy Grading at All 3 Centers A, General classification of screened images. B, Complete grading classification by retinopathy (R) and maculopathy (M) criteria. C, Grading classification of R grades. D, Grading classification of M grades. A indicates active; S, stable.

The combined prevalence of DR across all 3 sites is 33% (95% CI, 33%-33%). Prevalence rates of DR were highly variable across sites: the CEITC had the highest rate of DR (64.6% [95% CI, 64.0%-65.0%]), the NIO had a substantially lower rate (39.8% [95% CI, 39.0%-41.0%]), and the FDH, which was not an eye hospital, had only 13.0% (95% CI, 13.0%-14.0%) of patients with any DR (see Figure 1 for examples of DR grades). The Table presents the age, sex, and worst grade of retinopathy and maculopathy for the screened population. The combined prevalence of pre-PDR was 3.9%, the combined prevalence of PDR across all 3 sites was 7.8%, and the combined prevalence of maculopathy was 19.2% (Figure 2). The prevalence of pre-PDR was 9.2% at the CEITC, 1.3% at the FDH, and 3.3% at the NIO. The prevalence of PDR was 16.0% at the CEITC, 1.3% at the FDH, and 11.5% at the NIO. The prevalence of maculopathy was 34.1% at the CEITC, 5.4% at the FDH, and 30.3% at the NIO.

The odds ratio of DR in urban vs rural locations was 7.19 (95% CI, 6.90-7.53) for the CEITC or NIO compared with the FDH. The eTable in the Supplement presents the breakdown of the odds ratios of urban centers vs rural centers stratified by age group. Across all age groups, male sex was significantly associated with having DR (odds ratio, 1.99; 95% CI, 1.90-2.07) (R1, R2, R3, and M1; eTable in the Supplement). A logistic regression model was performed to determine the associations of age and sex with the likelihood of DR. The model was statistically significant (χ^2^ = 2072.9; *P* < .001). Male patients had an odds ratio of 1.76 (95% CI, 1.68-1.84) for DR compared with female patients (*P* < .001), and increasing age increased the likelihood of DR. The odds ratio analyses of sex across age groups are shown in the eTable in the Supplement.

**Figure 3.**
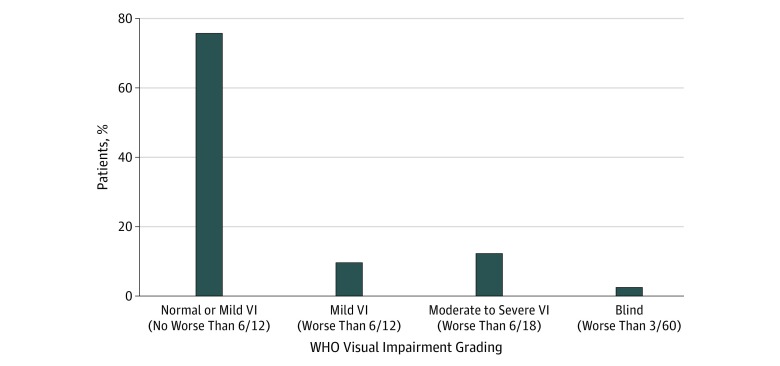
World Health Organization (WHO) Classification of Visual Impairment (VI) Across All 3 Screening Centers

**Figure 4.**
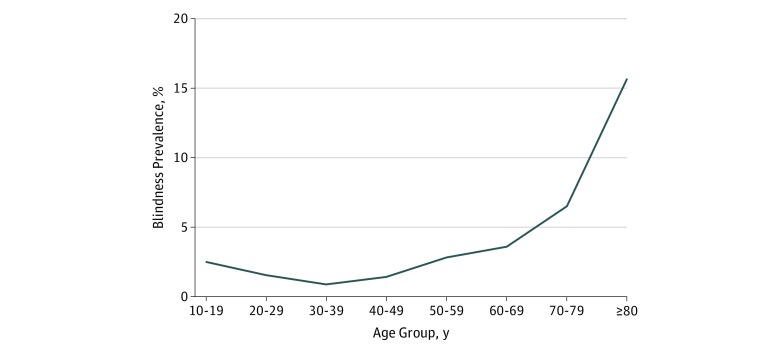
Prevalence of Blindness According to Age Group Among People With Diabetes Screened at 3 Centers in Bangladesh

The levels of visual impairment for various causes among patients with diabetes are shown in Figure 3. The rates of moderate visual impairment were 12.2% (combined), with subanalyses showing 11.6% for the CEITC, 10.3% for the FDH, and 16.6% for the NIO. The rates of blindness were 2.5% (combined), with subanalyses showing 3.3% for the CEITC, 2.2% for the FDH, and 2.4% for the NIO. Binocular blindness was more common with increasing age with the exception of the youngest age groups (Figure 4).

There was a statistically significant association between VA and DR grade as determined by 1-way analysis of variance (*F* = 1245.377; *P* < .001). A Tukey post hoc test revealed that individuals with all grades of DR (R1M0, R1M1, R2M1, R3M0, and R3M1) had significantly worse logMAR VA compared with those with normal results (R0M0) except for R0M0 vs R2M0. Within all grades of retinopathy, individuals with maculopathy (M1) had worse logMAR VA. Patients with ungradable fundus images had significantly worse logMAR VA compared with all other groups.

In the FDH and CEITC centers, 655 patients received laser photocoagulation, 725 patients received intravitreal eye injection therapy, and 54 patients underwent vitrectomy. In the NIO center, 982 patients received laser photocoagulation, 715 patients received intravitreal eye injection therapy, and 255 patients underwent vitrectomy. The higher number of patients who underwent vitrectomy at the NIO center reflects the more advanced vitreoretinal complications associated with PDR in their program.

Patients received macular laser for diabetic macular edema, panretinal laser treatment for PDR, and intravitreal injection for severe diabetic macular edema. Vitrectomy was provided at the CEITC and NIO for vitreoretinal complications of diabetes. For a short period, the laser machine was nonoperational and awaiting repair. Patients had their treatment delivered at private clinics during this interval.

A 4-person team was trained to provide diabetic counseling to increase awareness and improve eye health–seeking behavior among patients with diabetes. Clinic counselors, the health educator, and outpatient department physicians at all centers encouraged their patients with diabetes to undergo DR screening at least once in a year. They also motivated their patients to follow healthy practices for controlling their diabetes to help prevent adverse outcomes, including DR. Several diabetes and DR counseling videos were shown continuously for patients and caregivers in waiting rooms at all centers. Patients also received a variety of printed educational materials focusing on strategies to more effectively manage their diabetes, including healthy recipes and exercise logs; on the risks of vision loss due to diabetes; and on the need for annual eye examinations and DR screening.

## Discussion

We screened for DR using fundus photography in 2 eye centers (CEITC and NIO) and 1 diabetic hospital (FDH) and found a high prevalence of DR, with substantially higher rates in the eye centers. Male patients were at higher risk of prevalent DR than female patients across all age groups in the 3 centers, and DR was associated with older age, consistent with previous reports.^13,14,15^

Considering the large gap in care in Bangladesh, we focused primarily on the government system, which provides the most effective avenue to reach low-income patients in Bangladesh because government facilities are most widely used by low-income people. Bangladesh has one of the highest incidence rates of diabetes globally.^6,9,10^ We report a higher DR prevalence in the eye hospital clinics than in diabetic hospital clinics in Bangladesh. This finding may be associated with the higher affluence of urbanization in Bangladesh and the associated change in dietary habits that has increased the risk of diabetes, or it could be associated with the fact that those who present directly to eye hospitals have a higher likelihood of having eye problems. The prevalence of DR in the Bangladesh populations studied is similar to rates of DR in upper-middle-income countries and high-income countries such as the United Kingdom, the United States, and Singapore.^16,17,18^ The rates of maculopathy in Bangladesh were similar to the rates of diabetic macular edema in the United States.^19^ The similarity in high prevalence rates of DR and maculopathy between Bangladesh and upper-middle-income countries and high-income countries may be associated with the high incidence of diabetes and changing lifestyles in a transforming context, the higher incidence of childhood obesity, physical inactivity, and urbanization.^20,21^

The prevalence of blindness in Bangladesh was estimated in 2003 to be 1.53% (95% CI, 1.31%-1.75%) in individuals 30 years or older.^22^ The main cause of blindness was cataracts (80%), with a blindness rate of 1.4% in the CEITC and 1.1% in the NIO. The report estimated that 25% of patients with diabetes had DR, although DR was not associated with blindness or low vision.^22^ Nearly one-third of older adults have diabetes in Bangladesh. Our DR screening centers screened male and female patients in considerable numbers from both urban and rural areas, and the age groups were distributed across a broader range from 10 to 116 years. The rates of blindness in our study were more than twice those estimated in 2003, but the exclusive focus on those with diabetes likely explains this difference.

For DR screening to be sustainable, either government funding or cost recovery is needed. In our programs, an important reason for engagement of patients in the DR screening centers was the use of a partial cost recovery system. The model piloted in this program—namely, housing the DR screening in eye hospitals—reduces costs and increases the rate of identification of DR. The Bangladesh DR screening program uses an international-standard grading center with DR software, and the grading center reviews fundus photographs electronically transmitted from 3 screening centers. While patients wait, photographs are remotely reviewed by a team of graders, and recommendations for follow-up are relayed, enabling screening center staff to immediately advise on treatment plans, which reduces the risk that patients will not receive the information or treatment they need by failing to return for follow-up. The DR screening program has become sustainable for the host hospital centers, as evidenced in the CEITC and FDH centers, but further scaling is required across other regions in Bangladesh.

The Bangladesh National Eye Care plan has previously focused on screening by ophthalmologists,^22^ which is less cost-effective than screening with fundus cameras and a grading center and reaches fewer patients. Given the fact that most people with diabetes do not have DR, a practical approach is to have trained midlevel personnel (not ophthalmologists) grade fundus photographs initially and only refer those who have abnormalities for care. Screening, grading, and follow-up must be integrated because many individuals will not obtain treatment unless they are assisted in the process of obtaining care. Diabetic retinopathy screening centers based in eye hospitals have demonstrated that eye care facilities are also effective screening sites because patients are able to consult with a retina specialist and receive treatment in a fixed-location screening center. This factor reduces issues with patients failing to follow up on referrals and reduces travel time, which is key for low-income patients. Including eye care facilities in screening also dramatically increases the number of possible locations for screening services. If Bangladesh is to effectively address the increasing burden of diabetic eye disease, then screening services using a nonmedical workforce are recommended to reach all patients, as demonstrated to be effective in our programs, and should involve DR screening within diabetes hospitals and clinics in order to achieve optimal coverage.

### Limitations

We report a number of study limitations. Snellen VA was recorded and was converted into logMAR VA for analysis. The accuracy of an ungradable image diagnosis was not complete, and the documentation of cataracts as ocular comorbidity and underlying DR cause was suboptimal. However, missing DR grading outcomes were minimized because the screening DR grades were extracted from the electronic patient record and externally validated by a second data spreadsheet. In this project, we measured the key performance indicator of a first treatment episode (laser, intravitreal injection, or vitrectomy) after a positive retinopathy and maculopathy screening episode. Our screening programs did not follow up with the longer-term outcomes for patients after they received the primary treatment because this clinical treatment audit would remain under the auspices of the treating hospital eye center.

## Conclusions

There are several policy implications of our study for Bangladesh with respect to current practice for DR screening and prevalence of diabetes in the country. Diabetic retinopathy is recognized as a health priority in the government of Bangladesh’s National Eye Care Plan, overseen by the NIO.^23^ Diabetic retinopathy screening can be implemented successfully in Bangladesh; expanding such programs could prevent many cases of unnecessary blindness. The Bangladeshi government should identify and support centers that carry out this work. Our DR screening programs have provided strong evidence for future consideration of a national model for DR screening, grading, treatment, and prevention integrated into the government of Bangladesh’s National Eye Care Plan on a larger scale across the whole country.

The merits of scaling up the DR screening model include the fact that grading can be performed remotely and 1 site with expertise can be used by multiple locations. The costs of retinal photography have decreased, with many lower-cost devices now available. The major challenge is identifying committed organizations to take ownership of the expanded program and ensuring that those who require care will have coverage to allow for treatment. Identifying the best economic model to ensure success remains a challenge. The government of Bangladesh’s National Eye Care Plan is currently under review by a government-appointed committee, with Helen Keller International as a member of the task force.

## Notes

Supplement. **eTable.** Association of Urban and Rural Centers, Sex, and Age With Diabetic Retinopathy
