# Supplementary material for: Trends in Diabetic Retinopathy, Visual Acuity, and Treatment Outcomes for Patients Living With Diabetes in a Fundus Photograph–Based Diabetic Retinopathy Screening Program in Bangladesh
Source: JAMA Netw Open. 2019 Nov 27;2(11):e1916285. doi: 10.1001/jamanetworkopen.2019.16285 (PMC6902843; doi:10.1001/jamanetworkopen.2019.16285)
Supplement: Supplement. — eTable. Association of Urban and Rural Centers, Sex, and Age With Diabetic Retinopathy [file jamanetwopen-2-e1916285-s001.pdf]

Supplementary Online Content

Muqit MMK, Kourgialis N, Jackson-deGraffenried M, et al. Trends in diabetic retinopathy, visual acuity, and treatment outcomes for patients living with diabetes in a fundus photograph–based diabetic retinopathy screening program in Bangladesh. *JAMA Netw Open.* 2019;2(11):e1916285. doi:10.1001/jamanetworkopen.2019.16285

eTable. Association of Urban and Rural Centers, Sex, and Age With Diabetic Retinopathy

This supplementary material has been provided by the authors to give readers additional information about their work.

© 2019 Muqit MMK et al. *JAMA Network Open.*

eTable. Association of Urban and Rural Centers, Sex, and Age With Diabetic Retinopathy

	Age group			
	0-20	21-39	40-59	60+
OR* of Urban/rural developing DR†	14.76 (3.88-56.09)	8.42 (67.33-9.69)	6.64 (6.21-7.1)	5.98 (5.41-6.62)
OR* of Male/Female developing DR†	1.554 (0.532-4.540)	1.859 (1.629-2.121)	1.966 (1.853 -2.085)	1.7 (1.552-1.862)

Footnotes: *OR-odds ratio; †DR- diabetic retinopathy

© 2019 Muqit MMK et al. *JAMA Network Open.*
